# Metabolism in the Tumour-Bone Microenvironment

**DOI:** 10.1007/s11914-021-00695-7

**Published:** 2021-07-28

**Authors:** Jessica Whitburn, Claire M. Edwards

**Affiliations:** 1 Nuffield Dept. of Surgical Sciences, University of Oxford, Oxford, UK; 2 Nuffield Dept. of Orthopaedics, Rheumatology & Musculoskeletal Sciences, University of Oxford, Oxford, UK; 3 Botnar Research Centre, Old Road, University of Oxford, Oxford, OX3 7LD UK

**Keywords:** Cancer, Bone, Metabolism, Microenvironment, Metastasis, Glycolysis

## Abstract

**Purpose of Review:**

For solid tumours such as breast and prostate cancer, and haematological malignancies such as myeloma, bone represents a supportive home, where the cellular crosstalk is known to underlie both tumour growth and survival, and the development of the associated bone disease. The importance of metabolic reprogramming is becoming increasingly recognised, particularly within cancer biology, enabling tumours to adapt to changing environments and pressures. This review will discuss our current understanding of metabolic requirements and adaptations within the tumour-bone microenvironment.

**Recent Findings:**

The bone provides a unique metabolic microenvironment, home to highly energy-intensive processes such as bone resorption and bone formation, both of which are dysregulated in the presence of cancer. Approaches such as metabolomics demonstrate metabolic plasticity in patients with advanced disease. Metabolic crosstalk between tumour cells and surrounding stroma supports disease pathogenesis.

**Summary:**

There is increasing evidence for a key role for metabolic reprogramming within the tumour-bone microenvironment to drive disease progression. As such, understanding these metabolic adaptations should reveal new therapeutic targets and approaches.

## Introduction

The bone microenvironment provides a fertile soil for a number of different malignancies to home, grow and survive. This includes solid tumour metastases such as breast cancer and prostate cancer, haematological malignancies such as multiple myeloma and primary bone tumours such as osteosarcoma and Ewings sarcoma. Once within the bone, a myriad of cellular interactions drive both the growth and survival of the tumour cells and the development of the associated bone disease [1]. These reciprocal relationships underpin the aggressive nature of tumours within the bone, and were first proposed several decades ago, with the seminal discovery that tumour cells produce osteoclast-activating factors [2]. Over the years, advances in technology have dramatically increased our understanding of this cellular crosstalk, identifying multiple factors and mechanisms that drive disease progression. This has been translated to the clinic in the form of biomarkers and new treatment regimens. Over recent years, the significance of metabolic reprogramming in disease pathogenesis has become increasingly recognised, representing a new mechanistic avenue to advance our understanding of these devastating malignancies. In this review, we provide an overview of current research, revealing insights into metabolic reprogramming and metabolic crosstalk within the tumour-bone microenvironment.

## Cancer and Metabolism

The deregulation of cellular energetics is now thought of as one of the hallmarks of cancer, with major reprogramming of cellular energy metabolism required in order to support the functional requirements of malignant cells, such as continued proliferation [3]. It has long been recognised that many cancer cells undergo a metabolic reprograming of their glucose metabolism from oxidative phosphorylation to aerobic glycolysis, known as the Warbug Effect [4]. This is somewhat counterintuitive, as the generation of ATP from glycolysis is significantly poorer relative to mitochondrial oxidative phosphorylation; however, aerobic glycolysis does give cancer cells some advantages including the ability to live in conditions of fluctuating oxygen tension, acid production to promote tumour invasion, and the production of glycolytic intermediates that can be used for anabolic reactions. This increased dependence on glucose has been associated with multiple tumourigenic changes, such as oncogene activation and mutation of tumour suppressors [5]. There is a wealth of information regarding the metabolic plasticity and metabolic reprogramming of cancer cells, with alterations in multiple metabolic pathways working both alone and in concert to drive malignant progression [5]. Furthermore, there is increasing evidence for distinct metabolic changes during the metastatic process, with dynamic metabolic changes supporting tumour cells at each step of the metabolic cascade [6]. The importance of the tumour microenvironment has long been recognised in virtually all aspects of cancer biology, and metabolic reprogramming is no exception. It is now accepted that tumour cells communicate with their neighbouring cells in order to support their bioenergetic demands. This can take the form of competition, where tumour cells compete with surrounding cells for substrates such as nutrients, or more of a metabolic symbiosis, with crosstalk supporting the metabolic needs of both cell types [7]. Two of the most widely studied cells of the tumour microenvironment in the context of metabolic plasticity are stromal cells, or cancer-associated fibroblasts (CAFs) and adipocytes. CAFS, by their very nature, are altered by tumour cells, with evidence to suggest that metabolic reprogramming of CAFs can support their heightened proliferation [8, 9]. The concept of the ‘reverse Warburg effect’ has been proposed for some fibroblast-cancer interactions, where CAFs metabolise glucose by aerobic glycolysis, and export lactate and other energy-rich intermediates to cancer cells to fuel oxidative phosphorylation [10, 11]. Adipocytes have been shown to serve as a lipid source for cancer cells, releasing free fatty acids and facilitating increased lipid utilisation by cancer cells and often resulting in lipolysis of adipocytes [12]. In contrast to the primary tumour, metabolic crosstalk within the metastatic site is poorly understood, and the bone is no exception. However, cellular crosstalk is well known to drive the vicious cycle of bone metastases, and the metabolic changes associated with this represent a wealth of information and potential for therapeutic exploitation.

## Bone and Metabolism

The bone is a highly dynamic microenvironment, with a delicate balance between osteoclast and osteoblast activity underlying normal bone homeostasis. The process of osteoclastic bone resorption occurs rapidly and is highly energy-intensive, requiring the dissolving of bone mineral and degradation of collagen. Osteoclasts contain high numbers of mitochondria, supporting this high-energy requirement, and RANKL has been shown to stimulate mitochondrial biogenesis [13, 14]. Osteoclasts have been shown to be dependent upon glucose metabolism through both glycolysis and oxidative phosphorylation, with a key role for lactate production [15]. Lemma et al. demonstrate a distinction in energy requirements between osteoclast formation and activity, with osteoclast formation dependent upon oxidative phosphorylation and resorption dependent on glycolysis [16]. While the metabolic plasticity of osteoclasts is not fully understood, their importance as a highly active, energy-dependent cell within the bone microenvironment is clear [17]. Similar to osteoclasts, the importance of metabolism within cells such as osteoblasts, stromal cells and adipocytes, generated from skeletal stem cells, is emerging [18]. Bone formation is an energy-intensive and metabolically demanding process. Skeletal glucose uptake is high, and the glucose transporter GLUT 1 is required for proper osteoblast differentiation and bone formation [19]. The majority of glucose is metabolised to lactate through aerobic glycolysis, which can be regulated by a number of anabolic stimuli [20]. An increase in oxidative phosphorylation has also been observed during osteoblast differentiation [21]. The skeleton is primarily a hypoxic environment, and this is exacerbated by the presence of tumours. Hypoxia is well known to induce metabolic changes; however, the effect of hypoxia on normal and pathological bone homeostasis, and within the tumour-bone microenvironment, is well described elsewhere and will not form a focus of this review [22].

## Metabolism in the Tumour-Bone Microenvironment

### Prostate Cancer Bone Metastases

Prostate cancer bone metastases represent a final step of metastasis, associated with aggressive tumour growth and the development of a primarily osteoblastic bone disease. Interactions between prostate cancer cells and osteoblasts drive the increase in bone formation, and reciprocal interactions support the growth of the tumour [23]. Benign prostate epithelia has a unique metabolism with high levels of zinc blocking the TCA cycle allowing the excretion of high levels of citrate into semen. Zinc levels fall during malignant transformation, relieving the inhibition of the TCA cycle resulting in increased flow through the TCA cycle and increased oxidative phosphorylation (OXPHOS). As such, in contrast to many cell types that typically become less energy efficient as they undergo malignant change, prostate cancer cells actually become more energy efficient. While there are many studies examining prostate cancer metabolism in relation to the primary tumour, there is relatively little information regarding metabolic crosstalk specifically within the prostate cancer-bone microenvironment. To date, studies largely focus upon metabolic changes in castrate-resistant prostate cancer (CRPC), a late stage of prostate cancer associated with metastasis and treatment failure. Progression of prostate cancer is associated with an increase in de novo fatty acid synthesis which is independent from systemic lipid levels [24] with an increase in key enzymes in the lipogenic pathway, including fatty acid synthase (FASN) in metastatic CRPC. While not specific for bone metastasis, selective inhibition of FASN inhibits the growth of CRPC in vivo, with expression of FASN associated with the constitutively active AR variant AR-V7 [25•]. Analysis of CRPC patients revealed significant differences in lipidomic profiles, with a prognostic three-lipid signature associated with decreased survival [26]. Metabolomic analysis of prostate cancer bone metastasis is limited in patients, with an early study using gas chromatography-mass spectrometry demonstrating significant alterations in metabolites from patients with prostate cancer bone metastasis, including an increase in cholesterol [27].

Within the primary tumour, there is increasing evidence of metabolic crosstalk between prostate cancer cells and their surrounding stroma, including the reprogramming of CAFs towards a Warbug phenotype and the establishment of a lactate shuttle facilitated by monocarboxylate transporters [28, 29]. As such, it is highly possible that similar metabolic dysregulation may occur within the bone microenvironment, particularly since CAFS are known to play a role in prostate cancer bone metastasis. To date, only bone marrow adipocytes have been shown to induce metabolic changes within bone metastatic prostate cancer cells, promoting the Warburg phenotype with increased glycolysis and decreased oxidative phosphorylation, dependent upon HIF-1alpha activation. This switch towards the Warburg phenotype was associated with adipocyte lipolysis, suggesting that adipocyte-derived lipids may contribute to the metabolic reprogramming [30]. It is likely that other cells such as osteoblasts and stromal cells promote similar metabolic reprograming, and intriguing to speculate whether prostate cancer cells may support a change in osteoblastic metabolic requirements associated with osteosclerotic disease.

### Breast Cancer Bone Metastases

Breast cancer typically metastasises to sites including the lymph nodes, bones, liver, lung and brain, with each site offering a distinct supportive microenvironment. Using murine models of breast cancer metastasis, the metabolic profiles of breast cancer cells have been characterised, demonstrating distinct metabolic programmes between organ-selective metastatic breast cancer cells. Gene expression profiling revealed a distinct metabolic signature in liver-metastatic cells, as compared to those cells that metastasised to lung or bone, with OXPHOS favoured by lung and bone metastases and glycolytic reprogramming associated with liver metastasis [31]. More recent studies have associated lung and bone metastases with an increase in PGC-1alpha in both cell lines and patient samples [32]. Bone metastatic breast cancer cells have been found to have high levels of enzymes required for de novo serine synthesis [33], and to release large amounts of lactate [34]. L-serine and lactate have been found to play important roles in osteoclast differentiation and function, supporting a mechanism by which metabolic reprogramming of breast cancer cells may contribute to the associated osteolysis. Importantly, these metabolic changes offer an opportunity for pharmacological intervention, with inhibition of MCT1 found to block lactate-induced osteoclast activity.

### Multiple Myeloma

In contrast to solid tumours such as breast and prostate cancer, myeloma is a haematological malignancy that arises within the bone marrow; however, once within the bone, many of the cellular interactions driving tumour growth and bone disease are similar between these malignancies. The frequency with which bone marrow aspirates are taken during myeloma diagnosis and disease progression lends itself well to analyses such as metabolomics and lipidomics, with a number of studies demonstrating changes in the metabolic profiles of myeloma cells isolated from patients with multiple myeloma. One such study performed concurrent lipidomics and proteomics in myeloma cells isolated from newly diagnosed and relapsed patients, revealing a downregulation of phosphatidylcholines in relapsed myeloma [35]. Gonsalves et al. combined untargeted metabolite and targeted lipid profiling of bone marrow plasma from patients with myeloma and monoclonal gammopathy of undetermined significance (MGUS), revealing changes in amino acid profiles and a reduction in complex lipids in patients with myeloma, as compared to MGUS [36]. When patients with myeloma were compared to healthy donors, a perturbation in glutamate metabolism and carnitine synthesis was detected in bone marrow plasma, with results suggesting the potential for aspartate and threonine as plasma biomarkers [37].

Myeloma is largely regarded as a glycolytic tumour and our understanding of how metabolic changes can support myeloma growth and survival is rapidly increasing [38–40]. Parzych et al. elegantly demonstrate how the ATPase VCP/p97 and the amino acid–sensing kinase GCN2 work together to maintain myeloma cell metabolism and protein homeostasis [41•]. By infusing patients with MGUS, myeloma and healthy controls with 13C-labelled glutamine, an increase in glutamine anaplerosis into the TCA cycle was observed in plasma cells from patients with myeloma, providing insight into the malignant transformation from MGUS to myeloma [42•]. Increasing our understanding of the metabolic plasticity of myeloma cells will undoubtedly reveal new mechanisms for therapeutic intervention. Indeed, targeting glutamine metabolism has been shown to sensitise myeloma cells to the BH3 mimetic venetoclax (ABT-199) [43]. Metabolic reprogramming has been associated with drug resistance in myeloma cells, revealing HIF1alpha and LDHA as key targets to overcome drug resistance [44].

Myeloma is associated with an osteolytic bone disease, resulting from both an increase in osteoclastic bone resorption and a reduction in osteoblastic bone formation, presumably associated with changing energy requirements and highly dependent upon cellular interactions. Marlein et al. begin to address how interactions within the bone marrow microenvironment drive metabolic reprogramming within myeloma cells, demonstrating the intercellular transfer of mitochondria from bone marrow stromal cells to myeloma cells, which was dependent upon CD38 and resulted in a switch to oxidative phosphorylation [45••]. Importantly, blocking this transfer increased survival of myeloma-bearing mice in vivo. Intriguingly, a recent study has demonstrated how metabolic changes in myeloma cells can drive reciprocal changes in bone cells, with glutamine consumption by myeloma cells resulting in an increase in glutamine synthetase in bone marrow stromal cells, and an inhibition of their osteoblastic differentiation [45••]. The inhibition of osteoblast differentiation in glutamine-depleted mesenchymal stromal cells could be rescued by asparagine, providing a potential mechanism by which metabolic reprogramming in the myeloma-bone microenvironment may drive osteoblast suppression and may be alleviated by asparagine supplementation. Myeloma cells have also been shown to alter the metabolism of neighbouring bone marrow adipocytes, damaging the mitochondria of the adipocytes and so driving production of an abnormal cytokine profile and driving disease progression [46]. The idea that the bone marrow niche can control amino acid availability, secreting essential and non-essential amino acids has recently been highlighted in acute myeloid leukaemia, where stromal-derived aspartate was found to contribute to chemoresistance [47•]. The bone marrow niche is well known to support drug resistance in myeloma, and may be supported by similar changes in stromal and tumour metabolism.

### Primary Bone Cancers

Primary bone tumours such as osteosarcoma are also associated with metabolic changes, evident in both patient samples and in vitro experimental systems [48]. Increased understanding of the role of metabolic pathways in the biology of primary bone cancers has led to the identification of potential therapeutic targets, such as 3-phosphoglycerate dehydrogenase (PHGDH), the rate-limiting enzyme in the biosynthesis of serine from glucose. High levels of PHGDH are inversely correlated with survival in osteosarcoma patients, with inhibition of the enzyme effective in blocking proliferation of osteosarcoma cells [49]. Osteosarcoma is associated with increased aerobic glycolysis, regulated by multiple factors including microRNAs, ROCK and the transcription factor ELK1 [50–52]. Targeting glycolysis by inhibition of lactate dehydrogenase has been found to be highly effective in preclinical models of Ewing sarcoma by Yeung et al. employing a novel class of LDH inhibitors [53]. It is clear that by increasing our understanding of the metabolic requirements of such primary bone tumours, novel therapeutic targets will be revealed to combat these malignancies.

## Summary

At present, our understanding of exactly how the metabolic crosstalk between tumour cells and the bone microenvironment drives disease progression is limited. What is abundantly clear is that the key clinical features of bone metastases and malignancies such as multiple myeloma, namely tumour growth within bone and the development of a destructive bone disease, are both energy-intensive processes, likely dependent upon metabolic reprogramming of both tumour cells and bone cells for successful disease progression. In addition to tumour growth and bone disease, tumour dormancy is increasingly recognised as a key step in disease progression, with the activation of tumour cells from a dormant state driven at least in part by the microenvironment, and it is intriguing to speculate how metabolic plasticity may contribute. As such, understanding exactly how the metabolic requirements alter during disease will undoubtedly reveal new ideas and approaches to disrupt the symbiotic relationship between tumour and bone.
